# Mutation screen in the GWAS derived obesity gene *SH2B1* including functional analyses of detected variants

**DOI:** 10.1186/1755-8794-5-65

**Published:** 2012-12-27

**Authors:** Anna-Lena Volckmar, Florian Bolze, Ivonne Jarick, Nadja Knoll, André Scherag, Thomas Reinehr, Thomas Illig, Harald Grallert, Heinz-Erich Wichmann, Susanna Wiegand, Heike Biebermann, Heiko Krude, Pamela Fischer-Posovszky, Winfried Rief, Martin Wabitsch, Martin Klingenspor, Johannes Hebebrand, Anke Hinney

**Affiliations:** 1 Department of Child and Adolescent Psychiatry, University Duisburg-Essen, Virchowstr. 171, D 45147, Essen, Germany; 2 Molecular Nutritional Medicine, Else Kröner-Fresenius Center and Research Center for Nutrition and Food Science, Technical University Munich, München, Germany; 3 Institute of Medical Biometry and Epidemiology, University of Marburg, Marburg, Germany; 4 Institute of Medical Informatics, Biometry and Epidemiology, University of Duisburg-Essen, Essen, Germany; 5 Vestische Children's Hospital Datteln, University of Witten/Herdecke, North Rhine-Westphalia, Germany; 6 Institute of Epidemiology, Helmholtz-Zentrum, Munich, Germany; 7 Hannover Unified Biobank, Hannover Medical School, Hannover, Germany; 8 Institute of Experimental Pediatric Endocrinology, Charité, Berlin, Germany; 9 Division of Pediatric Endocrinology and Diabetes, Department of Children and Adolescent Medicine, University of Ulm University Medical Center, Ulm, Germany; 10 Division of Clinical Psychology, University of Marburg, Marburg, Germany

**Keywords:** SH2B1, Obesity, BMI, rs7498665, Mutation screen

## Abstract

**Background:**

The *SH2B1* gene (Src-homology 2B adaptor protein 1 gene) is a solid candidate gene for obesity. Large scale GWAS studies depicted markers in the vicinity of the gene; animal models suggest a potential relevance for human body weight regulation.

**Methods:**

We performed a mutation screen for variants in the *SH2B1* coding sequence in 95 extremely obese children and adolescents. Detected variants were genotyped in independent childhood and adult study groups (up to 11,406 obese or overweight individuals and 4,568 controls). Functional implications on STAT3 mediated leptin signalling of the detected variants were analyzed *in vitro*.

**Results:**

We identified two new rare mutations and five known SNPs (rs147094247, rs7498665, rs60604881, rs62037368 and rs62037369) in *SH2B1*. Mutation g.9483C/T leads to a non-synonymous, non-conservative exchange in the beta (βThr656Ile) and gamma (γPro674Ser) splice variants of *SH2B1*. It was additionally detected in two of 11,206 (extremely) obese or overweight children, adolescents and adults, but not in 4,506 population-based normal-weight or lean controls. The non-coding mutation g.10182C/A at the 3’ end of *SH2B1* was only detected in three obese individuals. For the non-synonymous SNP rs7498665 (Thr484Ala) we observed nominal over-transmission of the previously described risk allele in 705 obesity trios (nominal p = 0.009, OR = 1.23) and an increased frequency of the same allele in 359 cases compared to 429 controls (nominal p = 0.042, OR = 1.23). The obesity risk-alleles at Thr484Ala and *β*Thr656Ile/*γ*Pro674Ser had no effect on STAT3 mediated leptin receptor signalling in splice variants *β* and *γ*.

**Conclusion:**

The rare coding mutation *β*Thr656Ile/*γ*Pro674Ser (g.9483C/T) in *SH2B1* was exclusively detected in overweight or obese individuals. Functional analyzes did not reveal impairments in leptin signalling for the mutated SH2B1.

## Background

A large-scale genome-wide association study (GWAS) meta-analysis including a total of 249,796 individuals of European ancestry confirmed 14 known obesity-susceptibility loci and newly identified 18 genetic loci associated with body mass index (BMI). One of the re-identified single nucleotide polymorphisms (SNPs) is located near the Src-homology 2B adaptor protein 1 (*SH2B1*) gene (rs7359397) [1]. Association with obesity was also shown for a coding SNP in *SH2B1* (rs7498665: g.8164A/G, Thr484Ala; [2,3]). Linkage disequilibrium between rs7359397 and the coding SNP rs7498665 is high (r^2^ = 0.965, D’ = 1; HapMap, http://hapmap.ncbi.nlm.nih.gov/). Both SNPs are located within a large linkage disequilibrium (LD) block. A region of more than 500 kb upstream and 150 kb downstream of *SH2B1* is flanked by recombination peaks of 37 cM/Mb or 36 cM/Mb, respectively (SNP Annotation and Proxy Search SNAP, see Additional file 1: Figure S1). The association of increased BMI with *SH2B1* SNP (rs7359397 and rs7498665) alleles has been robustly replicated in e.g. (i) 4,923 Swedish adults [4], (ii) in 12,462 individuals from the German MONIKA/KORA study [5], and (iii) in 1,045 obese adults and 317 healthy lean individuals from Belgium [6].

A deletion of ~200 kb covering *SH2B1* was recently shown to be associated with severe early-onset obesity [7], whereas the corresponding reciprocal duplication was associated with leanness [8]. Additionally, a larger interspersed deletion extending through a 593 kb region on chromosome 16p11.2-p12.2 covering *SH2B1* has been associated with developmental delay, feeding difficulties, dysmorphic facial features, and obesity [9,10]. Bochukava et al. [7] screened the coding region of *SH2B1* for causal mutations by re-sequencing of 500 early onset severely obese children of the Genetics of Obesity Study (GOOS). The investigators detected SNP rs7498665 (Thr484Ala [7]); rare variants were not identified. Evidence in humans and from animal models suggests that *SH2B1* is a likely obesity gene. In humans, the SH2B1 protein increase serum leptin levels and whole body fat mass in females [11]. The influence of *SH2B1* variants on the distribution of body fat and the amount of visceral adipose tissue is still under discussion [4,12,13]. With regard to animal models, *Sh2b1* null mice show a phenotype of obesity, hyperlipidemia, leptin resistance, hyperphagia, hyperglycaemia, insulin resistance and glucose intolerance [14]. This phenotype was consistent when the knockout was regionally limited to hypothalamic neurons [15] and functionally limited to induced mutations in the Src-homology 2 (SH2) and pleckstrin homology (PH) domains [16]. Selective rescue in neurons eliminated both obesity and the insulin resistance phenotype [16]. Additional evidence for an involvement of Sh2b1 in the regulation of energy homeostasis is derived from expression analyses in mice and rats. In DIO (diet-induced obese) rats, fed a high fat diet, the expression of Sh2b1 in hypothalamus was decreased [17], while in mice on a high fat and high carbohydrate diet, Sh2b1 expression increased in the same tissue [18].

We initially detected transmission disequilibrium for a SNP in the vicinity of *SH2B1* (rs2008514) in 705 obesity trios (p =0.0094 of TDT)*.* The SNP is a proxy of rs7359397, which lit up in large scale GWAS [1]. We screened the coding region of *SH2B1* for (infrequent) mutations in 95 extremely obese children and adolescents. For the GWAS-derived gene for type 2 diabetes mellitus melatonin receptor 1B (*MTNR1B*) it was recently shown that a number of rare to infrequent mutations can be detected in the respective patients [19]. In addition, based on the evidence for involvement of SH2B1 in energy homeostasis, rare coding variants in the gene could potentially result in monogenic obesity. Subsequently, we assessed association of the identified variants to obesity in independent study groups. *In vitro* analyzes of the impact on leptin receptor signalling for the detected variants ensued.

## Material & methods

### Study groups

**Figure 1:**
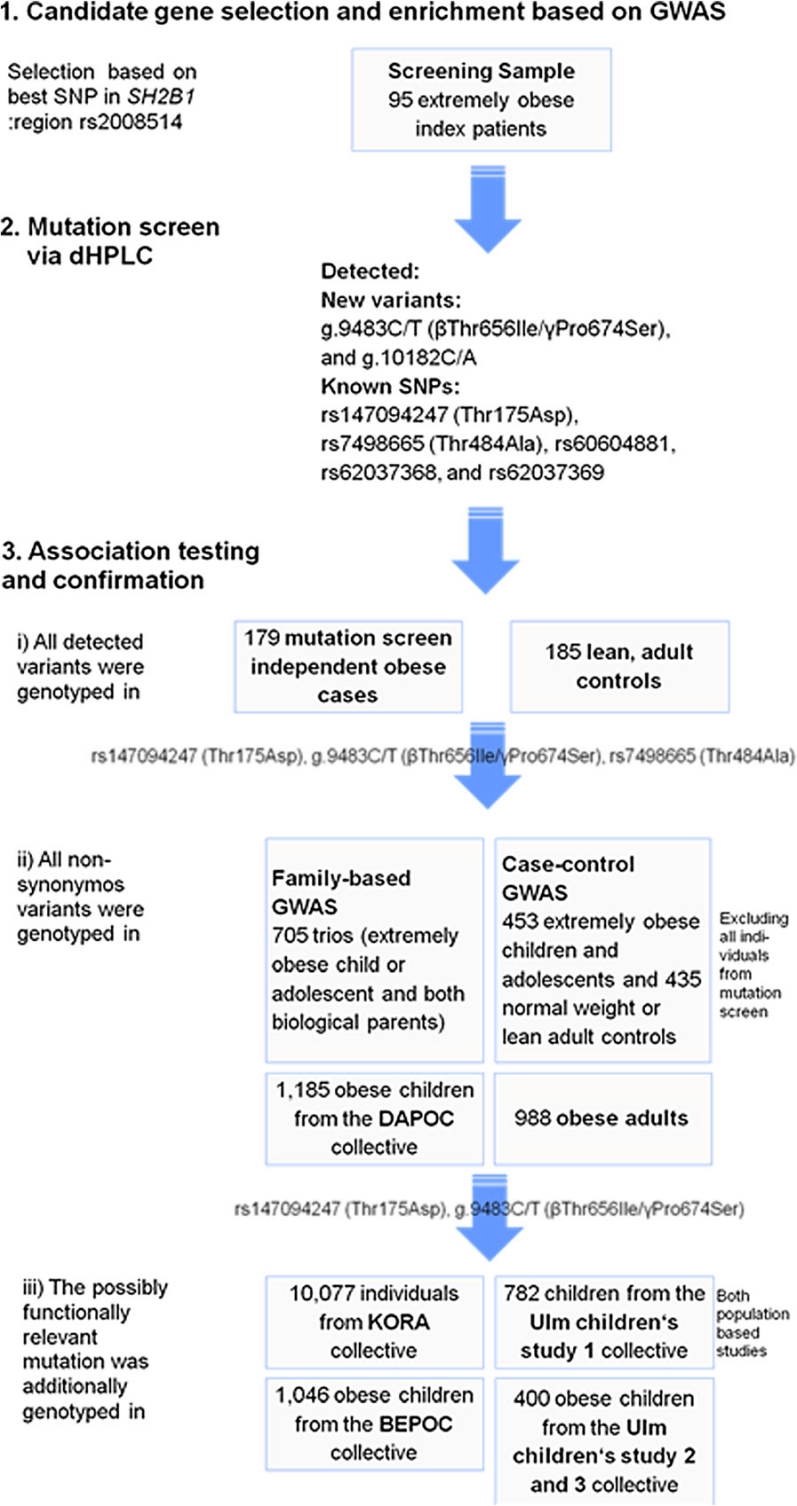
**Flow chart for experimental setup.** GWAS: genome-wide association study; SNP: single nucleotide polymorphism; dHPLC: denaturating high performance liquid chromatography; DAPOC: Datteln Paediatric Obese Cohort (Reinehr et al. 2007); KORA: Cooperative Health Research in the Region of Augsburg (Wichmann et al. 2005); Ulm children’s study 1: Ulm Research on Metabolism, Exercise and Lifestyle in Children (Nagel et al. 2009); BEPOC: Berlin Paediatric Obese Cohort (Bau et al. 2009); Ulm children’s study 2 and 3: Ulm Paediatric Obese Cohort A and B (INSULA) (Wabitsch et al. 2004).

An overview of the ten study groups is given in Table 1, details have been described previously [20,21]. The selection of individuals for the mutation screen was based on genotypes at SNP rs2008514 (proxy of rs7359397) in the vicinity of *SH2B1*. In total, we analyzed 95 individuals, 90 of whom were likely enriched for the presence of mutations in *SH2B1.* The other five individuals are heterozygous carriers of a deletion at chr16p11.2 which does not harbor *SH2B1*[10]. These extremely obese patients (offspring) from the family-based GWAS sample were homozygous for the risk allele T of rs2008514 and had at least one heterozygous parent, thus substantially contributing to the observed over-transmission of the rs2008514 T-allele. Association to obesity of detected variants was analyzed in three steps (Figure 1):

i. Association testing: All detected variants were genotyped in a sample of 179 extremely obese (age- and sex-adjusted BMI percentile ≥ 99^th^; [22]) children or adolescents and 185 lean adult (age- and sex-adjusted BMI percentile 「 15^th^; [22]) controls. Basic phenotypical characteristics are given in Table 1. Individuals were independent of the mutation screening sample and were part of our case–control GWAS sample (Genome-Wide Human SNP Array 6.0, see [20,21]).

ii. Further exploration of non-synonymous variants: The three non-synonymous variants (rs147094247: g.2749C/A - Thr175Asp; rs7498665: g.8164A/G - Thr484Ala; g.9483C/T - βThr656Ile/γPro674Ser) were additionally genotyped in the remaining individuals of the family-based sample and the cases and controls of our GWAS sample, who were not screened for mutations (see Mutation Screen section) and in 988 obese adults [23] as well as in 1,185 independent obese children or adolescents of the ‘Datteln Paediatric Obese Cohort’ (DAPOC [24]; Table 1).

iii. Additional exploration of rare non-synonymous variants: The two coding mutations without previously shown association to obesity βThr656Ile/γPro674Ser and rs147094247 - Thr175Asp - were additionally genotyped in three independent study groups comprising obese children and adolescents (‘Berlin Paediatric Obese Cohort’ — BEPOC; n = 1,046 [25]; Ulm Children's Study 2 and 3; n = 271 and 129, respectively [26]; Table 1) and in two independent population-based cohorts, the ‘Cooperative Health Research in the Region of Augsburg’ (KORA; n = 10,077 [27]) cohort of adults, and the ‘Ulm Children's Study 1’ (n = 782 [28]) study group of children and adolescents (Table 1).

**Table 1:** Phenotypic description of analyzed study groups

**Sample**^**a**^	**status**	**n**	**% male [%]**	**age [mean ± SD]**	**BMI [mean ± SD]**	**BMI SDS**^**b**^**[mean ± SD]**
**Mutation Screen**	Cases	95	48.42	13.43 ± 3.37	31.87 ± 5.04	4.10 ± 1.71
**S**_**I**_**: family-based GWAS**	Cases	705	45.11	13.44 ± 3.02	32.02 ± 5.82	4.23 ± 1.96
	Parents	1,410	50.00	42.54 ± 6.02	30.28 ± 6.33	1.65 ± 1.84
**S**_**II**_**: case–control GWAS**	Cases	453	42.60	14.37 ± 3.75	33.15 ± 6.68	4.51 ± 2.15
	Controls	435	39.08	26.08 ± 5.75	18.09 ± 1.14	−1.45 ± 0.34
**S**_**III**_**: subset of case–control GWAS (for association testing)**	Cases	179	49.16	14.27 ± 2.39	35.56 ± 6.13	5.3 ± 2.09
	Controls	185	55.68	25.56 ± 3.94	18.39 ± 1.09	−1.47 ± 0.33
**S**_**IV**_**: obese adults**	Cases	988	37.25	47.17 ± 14.23	35.70 ± 5.43	3.22 ± 1.66
**S**_**V**_**: DAPOC**	Cases	1,185	44.22	10.72 ± 2.78	27.69 ± 5.11	3.07 ± 1.56
**S**_**VI**_**: KORA**	Cases	6.633	56,43	56.62 ± 13.13	29.42 ± 3.70	1.19 ± 1.09
	Controls	3.444	36,76	47.18 ± 13.37	22.72 ± 1.68	−0.53 ± 0.51
**S**_**VII**_**: Ulm children’s study 1**	Cases	97	57.73	7.57 ± 0.42	20.68 ± 1.71	1.06 ± 0.46
	Controls	685	54.31	7.56 ± 0.42	15.63 ± 1.38	−0.25 ± 0.37
**S**_**VIII**_**: BEPOC**	Cases	1,046	47.99	10.89 ± 3.57	29.46 ± 5.91	3.54 ± 1.82
**S**_**IX**_**: Ulm children’s study 2**	Cases	271	51.29	11.07 ± 3.69	29.75 ± 6.04	3.62 ± 1.88
**S**_**X**_**: Ulm children’s study 3**	Cases	129	43.41	14.90 ± 1.84	40.45 ± 8.00	7.05 ± 2.92

^a^Mutation Screen sample: part of the family-based and the case–control GWAS samples’ cases; Family-based GWAS sample: 705 index patients with early-onset extreme obesity and their biological parent; case–control GWAS sample: GWAS of early-onset extremely obese children and adolescents in comparison to lean, adult controls; case–control sample for association testing: early-onset extremely obese children and adolescents in comparison to lean, adult controls; independent of initial screening sample; subset of cases-control GWAS sample; DAPOC: Datteln Paediatric Obese Cohort (27); KORA: Cooperative Health Research in the Region of Augsburg (30); Ulm children’s study 1: Ulm Research on Metabolism, Exercise and Lifestyle in Children (31); BEPOC: Berlin Paediatric Obese Cohort (28); Ulm children’s study 2 and 3: Ulm Paediatric Obese Cohort A and B (INSULA) (29).

^b^Calculation of the BMI SDS values has been based on population reference values following the National Nutrition Survey I (25).

In all samples, body mass index (BMI in kg/m^2^) was assessed and age- and sex-specific percentile criteria with regard to the German population at the time of sample recruitment (S_I_, S_II_, S_III_, S_IV_, S_VI_[22]) were used to define overweight or obese cases. Samples were divided into cases (adults: BMI ≥ 25, children and adolescents: ≥ 90^th^ BMI percentile (http://www.mybmi.de)) and controls (adults: BMI ≤ 25, children and adolescents: BMI ≤ 90^th^ percentile (http://www.mybmi.de)). Written informed consent was given by all participants and in case of minors by their parents. These studies were approved by the Ethics Committees of the respective Universities and Institutions and were performed in accordance with the *Declaration of Helsinki*.

### Mutation screen

The coding region of *SH2B1,* located at chr16: 28,858,010 - 28,885,533 (hg18/ NCBI 36), was screened for mutations by denaturating high pressure liquid chromatography (dHPLC, WAVE, Transgenomics) as described previously [29]. Accuracy of dHPLC is similar or even higher than sequencing [30]. To enhance the sensitivity of detection of homozygous mutation carriers, DNA of an individual with wild type genotype (re-sequenced) was added to each sample prior to PCR amplification. All samples with deviant dHPLC patters were re-sequenced. Detailed information regarding used temperatures and primers for PCR and dHPLC analysis can be obtained from the authors. All PCR amplicons with dHPLC patterns deviant from the wild-type pattern were re-sequenced as described previously [31]. At least two experienced individuals independently assigned the genotypes; discrepancies were solved either by reaching consensus or by re-genotyping.

### Genotyping

The identified variants in *SH2B1* were genotyped in larger study groups using either restriction fragment length polymorphism (RFLP) or TaqMan Assays (detailed information can be obtained from the authors). For rs147094247 (Thr175Asp) and the new coding mutation in fragment 9 (g.9483C/T *β*Thr656Ile/*γ*Pro674Ser), custom TaqMan assays were designed (SH2B1_2I_MUT, Assay ID: AHCS0BY, and Frag9_mut1, Assay ID: AHMSHDX, respectively, both Applied Biosystems). At least two experienced individuals independently assigned the genotypes; discrepancies were solved either by reaching consensus or by re-genotyping.

### Statistics

Allele and genotype distributions of all detected variants did not deviate from Hardy-Weinberg equilibrium. To analyze the obesity association of all variants, Fisher’s exact test (allelic association) was calculated with PLINK [32]. Population-based samples were divided into cases (BMI ≥ 90^th^ percentile) and controls (BMI 「 90^th^ percentile). For rs7498665 an asymptotic, 2-tailed p-value for the transmission disequilibrium test (TDT) was additionally calculated with PLINK. If not stated otherwise, all p-values are asymptotic, two-sided and not corrected for multiple testing.

### Functional *in silico* analyzes

To determine the potential alteration in gene expression, all mutations were analyzed for loss or gain of cryptic splice sites, transcription factor binding sites and gain or loss of o-glycosilation sites. Prediction of possible impact of amino acid exchange on structure and function of SH2B1 was done by PolyPhen-2 [33], SNAP [34], PMUT [35], and MutationTaster [36]. Detailed description of used tools can be found in the Additional file 1: Supplementary materials. Conservation was analyzed by aligning sequences of 21 species in total (21 α, eight β and six γ sequences, comprehensive list in Additional file 1: Supplementary materials).

### Functional *in vitro* analyzes: STAT3 mediated leptin receptor signalling

The effect of SH2B1 harboring the infrequent alleles of Thr484Ala and *β*Thr656Ile/*γ*Pro674Ser on leptin receptor activity was determined with a quantitative reporter gene assay (adapted from [37]). HEK293 cells were transiently transfected with the murine long form of the leptin receptor (*Lepr-b)* in pcDNA3.1, a signal transducer and activator of transcription 3 (*STAT3*) responsive *Photinus* luciferase construct (pAH32), a constitutive *Renilla* luciferase expression vector for data normalization (phRG-b, Promega) and human *SH2B1* splice variants beta and gamma with and without the mutations in pCMV-XL5 expression vectors (*Lepr-b* and pAH32 were kindly provided by Rosenblum et al. [37]). For control empty pcDNA3 expression vector was transfected instead of SH2B1. After stimulation with mouse leptin (concentrations 0, 0.5, 1, 5 , 10, 50, 100, 500 ng/ml), the STAT3 reporter construct led to luciferase expression measured with the Dual–Luciferase Reporter Assay system according to manufactures’ instruction (Promega). Dose–response curves, EC50 and Emax values were calculated by Graph Pad Prism.

## Results and discussion

We performed a mutation screen of the coding region of *SH2B1* in 95 extremely obese children and adolescents. We identified two unknown mutations and five known SNPs in *SH2B1* (Table 2). All detected variants were followed up in a small, independent case–control sample, only non-synonymous variants were additionally genotyped in further independent study groups.

**Table 2:** **Minor allele frequencies of the detected SNPs and mutations in *SH2B1 *excluding the screening group**

			**Genotypes cases**				**Genotypes controls**						
**Position**	**rs-Number**	**Amino acid exchange**	**11**	**12**	**22**	**Minor allele frequency cases [%]**	**11**	**12**	**22**	**Minor allele frequency controls [%]**	**Odds Ratio**^**d**^	**95% Confidence Interval**	**Nominal p-value**
g.2749C/A	rs147094247	Thr175Asp	11, 257	11	0	0.05	4,511	1	0	0.01	4.4	0.57 - 34.13	0.199^c^
g.8164A/G	rs7498665	Thr484Ala	512	1,526	1,101	40.62	58	195	181	35.83	1.2	1.06-1.42	**0.007**^a^
g.8250C/T	rs60604881		70	87	22	36.59	73	75	37	40.27	0.86	0.63 - 1.15	0.323^b^
g.8738A/G	rs62037368		176	2	0	0.56	182	3	0	0.81	0.69	0.11 - 4.16	1^b^
g.8764C/T	rs62037369		65	81	32	40.73	79	82	23	34.78	1.29	0.95 - 1.74	0.107^b^
g.9483C/T		βThr656Ile, γPro674Ser	11,206	2	0	0.01	4,506	0	0	0.00	NA	NA	1^c^
g.10182C/A			178	1	0	0.28	184	0	0	0.00	NA	NA	0.493^b^

^a^ genotyped in 3,230 obese cases and 439 lean controls.

^b^ genotyped in a total of 179 obese cases and 185 lean controls.

^c^ genotyped in a total of 11,406 obese and overweight cases and 4,568 (mainly population based) controls.

^d^ Odds ratio is given with respect to the minor allele.

### New infrequent variant *β*Thr656Ile/*γ*Pro674Ser

A new mutation at position g.9483 (C/T) of *SH2B1* results in a non-synonymous, non-conservative exchange in two of the three human splice variants (β and γ) of SH2B1. Due to a shifted reading frame for the two splice variants, the mutation results in two different non-synonymous, non-conservative exchanges (*β*Thr656Ile or *γ*Pro674Ser) in the β or γ splice variants, respectively. The *β*Thr656Ile/*γ*Pro674Ser mutation was not detected in an independent sample of 179 extremely obese cases and 185 lean controls. As the mutation resulted in a non-synonymous amino acid exchange which was predicted to change protein structure (Table 3), we additionally genotyped a total of 11,029 (extremely) obese or overweight children, adolescents and adults and 4,321 controls (for children and adolescents BMI 「 90^th^ percentile, for adults BMI 「 25kg/m2) for this mutation. We detected two additional obese cases with this mutation and no mutation carrier among the controls. The extremely low frequency of the mutation limits the determination of an association to overweight and obesity (p = 1; Table 2). We calculated that the control group would need to include more than 545,757 individuals to reveal a p-value below 0.05 with statistical power above 80%, if the observed trend (only mutation carriers among the overweight or obese individuals) would remain stable. Both risk alleles (T-allele at g.9483C/T and G-allele at rs7498665) are potentially located on the same haplotype (as determined in one index patient and his mother who transmitted the haplotype; for the other carriers, full genotype information of both parents was not available). A founder effect of this mutation is likely.

**Table 3:** ***In silico *prediction of splice sites, transcription factor binding sites and o-glycosylation sites of detected variants in *SH2B1***

**PROGRAM**			**ESEfinder**	**ESRSearch**	**RESCUE_ESE**	**TFSearch**	**Consite**	**OGPET**
**SNPs**	**Amino acid changes**	**DNA position**	*Splice sites*	*Splice sites*	*Splice sites*	*transcription factor binding sites*	*transcription factor binding sites*	*O-glycosylation site prediciton [%]*
rs147094247	Thr175Asp	g.2749C/A	changed	changed	not changed	not changed	not changed	33.1829
rs7498665	Thr484Ala	g.8164A/G	changed	changed	not changed	not changed	not changed	---
rs60604881	---	g.8250C/T	changed	SRp40, FOX1-FOX2^a^	not changed	not changed	not changed	---
rs62037368	---	g.8738A/G	changed	not changed	not changed	not changed	COUP-TF, c-REL ^b^	---
rs62037369	---	g.8764C/T	changed	changed	not changed	GATA1, GATA2, MZF1^b^	not changed	---
g.9483C/T	βThr656Ile, γPro674Ser	g.9483C/T	changed	not changed	not changed	GATA1, GATA2 ^b^	not changed	53.5329
g.10182C/A	---	g.10182C/A	changed	PESS ^a^	not changed	not changed	not changed	---

^a^ particular changed predicted splice sites, ^b^ particular changed predicted transcription factor binding sites.

All three detected *β*Thr656Ile/*γ*Pro674Ser mutation carriers are female. The initially identified mutation carrier (a) of the screening sample (height 163 cm, weight 86.2 kg, BMI 32.44 kg/m^2^, age 12.7 years) as well as one mutation carrier (b) from the follow-up samples (height 142 cm, weight 53.2 kg, BMI 26.38 kg/m^2^, age 9.9 years) had a BMI > 99^th^ percentile. In both cases, the overweight or obese mother (BMI 25.76 kg/m^2^ and 32.61 kg/m^2^, respectively) transmitted the mutation to the extremely obese child. The third mutation carrier (c) had a BMI > 90^th^ percentile (height 130 cm, weight 31.5 kg, BMI 18.64 kg/m^2^, age 7.2 years). For this mutation carrier, genotypic information about the parents was not available (mother BMI 19.81 kg/m^2^, father BMI 26.7 kg/m^2^). Insulin levels were only available for one mutation carrier (b), whose level was in the normal range (9.4 mU/l). Additional family members were not available.

**Figure 2:**
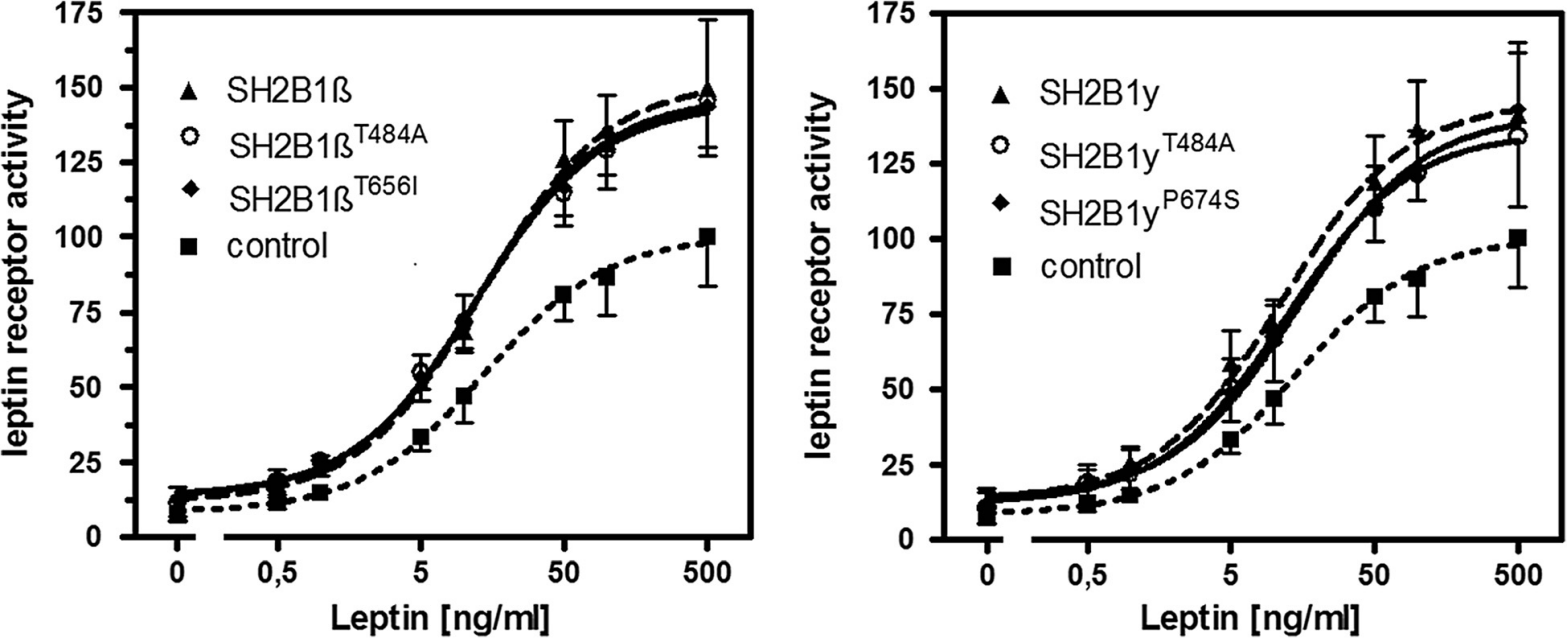
**Leptin receptor activity measured by STAT3 mediated luciferase response.** HEK293 cells (n = 8 separate experiments) were co-transfected with *LEPRb*, a STAT3 responsive element and *SH2B1* splice variants beta (left) and gamma (right) with and without the infrequent alleles at rs7498665 (Thr484Ala) and *β*Thr656Ile/*γ*Pro674Ser. The dose response curves depict leptin receptor activity after stimulation with leptin (exact values for each data point see Additional file 1: Table S2).

The amino acid exchanges in the β and γ splice variants (*β*Thr656Ile/*γ*Pro674Ser) are located outside the domain structure (self-dimerization, Pleckstrin-homology and SH2 domain; [34]), which is relevant for the function of SH2B1. We analyzed the impact of the variant rare allele of *β*Thr656Ile/*γ*Pro674Ser on leptin signalling *in vitro* via the STAT3 pathway. In both splice variants, *β*656Ile or *γ*674Ser showed unaltered leptin signalling (Figure 2, Additional file 1: Table S2).

While the *γ*Pro674Ser exchange in the *γ* splice variant is predicted to be neutral by *in silico* programs, the exchange of *β*Thr656Ile in the *β* splice variant was predicted to be “not neutral” (SNAP), “pathological” (PMUT) or “disease causing” (Mutation taster) in three of four programs (Additional file 1: Table S1). The exchange in the *β* splice variant would also destroy a predicted O-glycosylation site of the SH2B1 protein. Amino acid conservation was strong on both positions (86% for *β*Thr656Ile and 100% for *γ*Pro674Ser, Additional file 1: Table S1).

Previous *in vitro* data showed functional differences between *β* and *γ* SH2B1 variants: (a) It has been shown that the *β* splice variant is mainly expressed in the hypothalamus [16], a brain region known to be implicated in weight regulation. A Sh2b1*β* rescue was sufficient to prevent the Sh2b1 knockout phenotype in mice [19]. Leptin signalling is mediated by the interaction of SH2B1*β* and JAK2 [17]. The *β* splice variant of SH2B1 recruits insulin receptor substrates 1 and 2 (IRS1 and 2) to the LEPRb/JAK2 complex [38]. SH2B1*β* enhances JAK2 activity and promotes the activation of several downstream networks like STAT3 and phosphatidylinositol (PI) 3-kinase pathways [18,39]. Two *in silico* analyzes predicted an altered folding or function of SH2B1 *β*Thr656Ile (Additional file 1: Table S1). (b) The *γ* splice variant of SH2B1 is peripherally expressed. It interacts with Tyr1158 in the activation loop of the insulin receptor and prohibits dephosphorylation of IRS1 and IRS2 [40]. This interaction enhances insulin signalling and insulin receptor auto-phosphorylation, leading in turn to activation of downstream pathways [41]. While the three tyrosine motifs in the N-terminal part of SH2B1, which regulate interaction with the insulin receptor [42], are not directly affected by this exchange, it is possible that altered protein folding due to an non-conservative amino acid exchange in a highly conserved position prevents their phosphorylation.

### Coding GWAS derived SNP rs7498665

We confirmed that the described risk allele G at SNP rs7498665 [2,3] is associated with obesity in our 705 obesity trios (p = 0.009) and in a total of 3,139 independent cases and 434 controls (p = 0.007, odds ratio (OR) = 1.22, 95% confidence interval (CI) 1.06-1.42; Table 1). This coding SNP results in the non-synonymous, non-conservative amino acid exchange Thr484Ala in a slpice variant independent position with low conservation (5%, Additional file 1: Table S1). As the association with obesity was previously described for this SNP, we did not analyze further study groups (2–6). *In vitro* analyses revealed unaltered leptin signalling via STAT3 in both splice variants for the obesity risk allele at Thr484Ala (Figure 2). Both Emax and EC50 were non-significantly reduced (Figure 2, Additional file 1: Table S2).

Since the obesity risk allele at the GWAS derived polymorphism rs7498665 increases BMI by only approximately 0.15 BMI units (kg/m^2^) as calculated in a population of 125,931 European individuals [1], we expected only subtle functional alterations associated with the minor allele of this variant.

### Other genetic variants in *SH2B1*

The second newly detected mutation is located in the 3’ UTR at base pair position g.10182 (C/A). This non-coding variant was detected twice within the screening sample, and once in an obese case in the association testing step (Figure 1). The variant showed no association to obesity in a small case control comparison (p = 0.49; Table 2); *in silico* analyzes predicted a possible change in splice sites for this variant (Table 3).

Results for the four other identified SNPs were as follows: The third coding SNP rs147094247 leads to a non-synonymous, conservative exchange (Thr175Asp) at a conserved position (71%, Additional file 1: Table S1). No association to obesity (p = 0.199, odds ratio (OR) = 4.4, 95% confidence interval (CI) 0.57 - 34.13; Table 1) was found for this SNP in a sample of 11,268 obese and overweight cases and 4,512 lean or normal weight controls (mostly population based). For the non-synonymous SNP rs147094247 (Thr175Asp), *in silico* analyzes predict a neutral outcome for the altered amino acid (Additional file 1: Table S1).

For SNPs rs60604881 (p = 0.323, OR = 1.17, 95% CI = 0.87-1.58), rs62037368 (p = 1.00, OR = 1.45, 95% CI = 0.24-8.71) and rs62037369 (p = 0.107, OR = 1.29, 95% CI = 0.95-1.74) evidence for association to early onset extreme obesity could not be detected (Table 2). For all SNPs *in silico* analyzes predict splice site or transcription factor binding site changes (Table 3).

### Leptin signalling

The assay that measured STAT3 mediated leptin response successfully showed increased leptin response after co-transfection with wild type SH2B1 splice variants *β* and *γ*. This indicates that HEK293 cells and the STAT3 assay allow functional characterization of SH2B1. While in mice only the alpha splice variant was tested for leptin signalling [43], we observe an effect on leptin signalling for both other splice variants (*β* and *γ*) in our human cell system (Figure 2, Additional file 1: Table S2). The analysis of the impact of both SH2B1 variants on leptin receptor activity showed no significant reduction of STAT3 mediated signalling by the risk alleles at rs7498665 and *β*Thr656Ile/*γ*Pro674Ser. The non-significant decrease in EC50 and Emax for both tested variants in splice variants *β* and *γ* could indicate both gain of function and reduced function; the biological impact of both remains to be solved. If indeed a minor functional effect would be present, a much larger number of replicates would be necessary to establish a significant effect (e.g. about 2x270 replicates when using the Emax point estimates of SH2B1γ vs. SH2B1γP674S and their variances when applying Satterthwaite/Welch t-Test aiming at 80% power for two-sided α = 5%). Our results could, of course, indicate that the two variants are not functionally relevant. However, for a polygenic variant a large functional effect is rather unlikely. For example, the melanocortin 4 receptor gene (*MC4R*), a well known obesity gene, harbors two polymorphisms (Val103Ile and Ile251Leu) that are negatively associated with obesity [44,45]. Carriers of the minor alleles have a BMI approx. 0.5 BMI units lower than wild type carriers [44,45]. Initial *in vitro* assays did not show functional implications for the minor alleles of these SNPs (e.g. [44]), but when the number of different assays was increased, *in vitro* tests showed potential small gain of function for both minor alleles [46], which could explain the weight lowering effect of the variant. Hence we speculate that the functional effect of the analyzed *SH2B1* variants might become detectable when a battery of different functional tests is applied. Currently we have first hints that both variants are compatible with a slightly reduced function. In addition, with STAT3 mediated leptin signalling, we only tested one of the many potential interaction partners of SH2B1 in regulation of energy homeostasis. A potential additive effect of small functional changes in leptinergic and insulinergic signalling could result in stronger impact on body weight maintenance.

## Conclusion

A recent mutation screen in 300 children from the GOOS cohort which display insulin resistance in addition to obesity revealed three variants and one SNP that showed an effect on cell differentiation and migration, but with the exception of the frameshift variant Phe344fs no other functional deficiencies [47]. Comparable to our study, Doche et al. analyzed the impact of detected variants on janus kinase 2 (JAK2) phosphorylation with additional tests of insulin receptor substrate 2 (IRS2) phosphorylation and SH2B1 dimerization [47].

Given the low frequency of *β*Thr656Ile/*γ*Pro674Ser (g.9483C/T), this mutation cannot explain our positive TDT for rs2008514 with obesity. Adding the three rare mutations detected by Doche et al. which show low functional impact still leaves a large proportion of BMI association inexplicable [47]. The region around *SH2B1* on chr 16p11.2 shows low recombination rates for approximately 1Mb (chr16:28,177,800 shows a recombination peak of 37cM/Mb and chr16:28,944,400 a recombination peak of 36 cM/Mb; HapMap, http://hapmap.ncbi.nlm.nih.gov/), implicating a large region with high linkage disequilibrium. The area tagged by both BMI associated SNPs (rs7498665 and rs7359397 [1-3]) covers 17 genes (compare Additional file 1: Figure S1). Hence, relevant mutations in one of the remaining 16 genes might account for a larger proportion of the GWAS results. Alternatively, genetic variation outside of the *SH2B1* coding region with a regulatory effect on this gene explains the association in functional terms. Guo et al. recently showed an intronic SNP in SH2B1 (rs4788099) which regulated mRNA expression of nearby genes *Tu translation elongation factor, mitochondrial* (*TUFM)*, *coiled-coil domain containing 101* (*CCDC101)*, *Homo sapiens spinster homolog 1* (*SPNS1)*, *sulfotransferase family, cytosolic, 1A, phenol-preferring, member 1* (*SULT1A1)* and *sulfotransferase family, cytosolic, 1A, phenol-preferring, member 4* (*SULT1A4)* in B cells and monocytes [48]. This is in concordance with findings by Gutierrez-Aguilar et al. who reported differential regulation of Sh2b1, Tufm and Sult1a1 in rats fed a high fat diet [17].

In conclusion, the rare allele of the variant *β*Thr656Ile/*γ*Pro674Ser in SH2B1 was found exclusively in three overweight or obese children but not in normal-weight or underweight controls. Our findings suggest that this new rare mutation predisposes to increased BMI, possibly related to decreased leptin signalling. Further studies are warranted to investigate the functional impact of the mutation for both affected splice variants on the interaction of SH2B1 effector systems (e.g. leptin and insulin receptors), which play a major role in energy homeostasis.

## Abbrevations

BEPOC: Berlin Paediatric Obese Cohort; BMI: Body mass index; CCDC101: Coiled-coil domain containing 101; DAPOC: Datteln Paediatric Obese Cohort; DHPLC: Denaturating high performance liquid chromatography; DIO: Diet induced obese; GWAS: Genome-wide association study; GOOS: Genetics of Obesity Study; IRS2: Insulin receptor substrate 2; JAK2: Janus kinase 2; KORA: Cooperative Health Research in the Region of Augsburg; LEPRB: Long form of the leptin receptor; MC4R: Melanocortin 4 receptor; MTNR1B: Melatonin receptor 1b; PCR: Polymerase chain reaction; PH: Pleckstrin homology; RFLP: Restriction fragment length polymorphism; SH2B1: Src-Homology 2B adaptor protein 1; SH2: Src-homology 2; SNP: Single nucleotide polymorphism; SPNS1: Homo sapiens spinster homolog 1; SULT1A1: Sulfotransferase family, cytosolic, 1A, phenol-preferring, member 1; SULT1A4: Sulfotransferase family, cytosolic, 1A, phenol-preferring, member 4; STAT3: Signal transducer and activator of transcription 3; TDT: Transmission disequilibrium test; TUFM: Tu translation elongation factor, mitochondrial.

## Competing interests

Winfried Rief declares that he received financial support for presentations and for scientific advice from Astra Zeneca, Heel, and Berlin Chemie; he also declares that this did not influence the content of this manuscript. All other authors declare that they have no competing interests.

## Authors’ contributions

ALV participated in the design of the study, performed the mutation screen and some genotyping, was involved in the functional *in silico* and *in vitro* analyses, and drafted the manuscript. FB and MK established and performed the functional *in vitro* analyses. IJ and AS participated in the design of the study and performed the statistical analysis. NK, TI and HG genotyped individuals. TR, HEW, SW, HB, HK, PFP, WR, and MW recruited subjects and determined phenotype parameters. JH participated in the design of the study and writing of the manuscript. AH conceived of the study, its design and coordination and writing of the manuscript. All authors read and approved the final version of the manuscript.

## Pre-publication history

The pre-publication history for this paper can be accessed here:

http://www.biomedcentral.com/1755-8794/5/65/prepub

## Supplementary Material

Additional file 1 **Table 1.***In silico* functional prediction of detected non-synonymous mutations in *SH2B1.***Table2:** Parameters of leptin receptor activity measured by STAT3 mediated luciferase response. **Figure1:** Identified variants in the three splice variants (α, β and γ) of human *SH2B1. SH2B1* mRNA – coding parts as filled blocks – (Ensembl sequences α: ENST00000322610, β: ENST00000359285 and γ: ENST00000337120). The domain structure (Quian and Ginty 2001) with dimerization, Pleckstrin homology and SH2 domain is shown as underlying grey boxes. Positions of detected variants are marked with lines. Available rs-numbers, if applicable amino acid exchanges and minor allele frequencies in obese cases (MAF according to **Table1**) are given for each variant. Supplementary material: *In silico* analysis tool description. Supplementary **Figure2:** Regional association and linkage disequilibrium plot of 1000 genome project data centered to SNP rs7498665 (http://www.525broadinstitute.org/mpg/snap/). Displayed are recombination rate (blue), r² to rs7498665 (range of grey, increased intensity shows higher linkage) and genes in region. Dashed lines mark a region in high LD (r² > 0.8) with rs7498665. Gene abbreviations: *EIF3CL/EIF3C* (eukaryotic translation initiation factor 3), *CLN3* (ceroid-lipofuscinosis, neuronal 3), *APOB48R* (apolipoprotein B48 receptor), *IL27* (interleukin 27), *NUPR1* (p8 protein isoform a), *CCDC101* (coiled-coil domain containing 101), *SULT1A1* (sulfotransferase family, cytosolic, 1A, member 1), *SULT1A2* (sulfotransferase family, cytosolic, 1A,member 2), *ATXN2L* (ataxin 2 related protein isoform C), *TUFM* (Tu translation elongation factor, mitochondrial), *SH2B1* (SH2B adaptor protein 1 isoform 1), *ATP2A1* (ATPase, Ca++ transporting, fast twitch 1 isoform), *RABEP2* (rabaptin, RAB GTPase binding effector protein 2), *CD19* (CD19 antigen precursor), *NFATC2IP* (Nuclear factor of activated T-cells, cytoplasmic 2-interacting protein), *SPNS1* (spinster homolog 1 isoform 1), *LAT* (linker for activation of T cells isoform b). **Figure 3:** Conservation in C-terminal sequences of SH2B1 splice variants (β and γ). Boxes mark the position of exchange g.9483C/T (*β*Thr656Ile/*γ*Pro674Ser) in β and γ splice variants in several species. Click here for file

## References

[B1] Speliotes EK Willer CJ Berndt SI Monda KL Thorleifsson G Jackson AU Lango Allen H Lindgren CM Luan J Mägi R Randall JC Vedantam S Winkler TW Qi L Workalemahu T Heid IM Steinthorsdottir V Weedon MN Wheeler E Wood AR Ferreira T Weyant RJ Segrè AV Estrada K Liang L Nemesh J Park JH Gustafsson S Kilpeläinen TO Yang J Association analyses of 249,796 individuals reveal eighteen new loci associated with body mass index Nat Genet 2010 42 937 948 10.1038/ng.686 20935630 PMC3014648

[B2] Willer CJ Speliotes EK Loos RJ Li S Lindgren CM Heid IM Berndt SI Elliott AL Jackson AU Lamina C Lettre G Lim N Lyon HN McCarroll SA Papadakis K Qi L Randall JC Roccasecca RM Sanna S Scheet P Weedon MN Wheeler E Zhao JH Jacobs LC Prokopenko I Soranzo N Tanaka T Timpson NJ Almgren P Bennett A Six new loci associated with body mass index highlight a neuronal influence on body weight regulation Nat Genet 2009 41 25 34 10.1038/ng.287 19079261 PMC2695662

[B3] Thorleifsson G Walters GB Gudbjartsson DF Steinthorsdottir V Sulem P Helgadottir A Styrkarsdottir U Gretarsdottir S Thorlacius S Jonsdottir I Jonsdottir T Olafsdottir EJ Olafsdottir GH Jonsson T Jonsson F Borch-Johnsen K Hansen T Andersen G Jorgensen T Lauritzen T Aben KK Verbeek AL Roeleveld N Kampman E Yanek LR Becker LC Tryggvadottir L Rafnar T Becker DM Gulcher J Genome-wide association yields new sequence variants at seven loci that associate with measures of obesity Nat Genet 2009 41 18 24 10.1038/ng.274 19079260

[B4] Renström F Payne F Nordström A Brito EC Rolandsson O Hallmans G Barroso I Nordström P Franks PW GIANT Consortium Replication and extension of genome-wide association study results for obesity in 4923 adults from northern Sweden Hum Mol Genet 2009 18 1489 1496 10.1093/hmg/ddp041 19164386 PMC2664142

[B5] Holzapfel C Grallert H Huth C Wahl S Fischer B Döring A Rückert IM Hinney A Hebebrand J Wichmann HE Hauner H Illig T Heid IM Genes and lifestyle factors in obesity: results from 12,462 subjects from MONICA/KORA Int J Obes (Lond) 2010 34 1538 1545 10.1038/ijo.2010.79 20386550 PMC3251754

[B6] Beckers S Zegers D Van Gaal LF Van Hul W Replication of the SH2B1 rs7498665 Association with obesity in a Belgian study population Obes Facts 2010 4 473 477 22248999 10.1159/000335305 PMC6444515

[B7] Bochukova EG Huang N Keogh J Henning E Purmann C Blaszczyk K Saeed S Hamilton-Shield J Clayton-Smith J O’Rahilly S Hurles ME Farooqi IS Large, rare chromosomal deletions associated with severe early-onset obesity Nature 2010 463 666 670 10.1038/nature08689 19966786 PMC3108883

[B8] Jacquemont S Reymond A Zufferey F Harewood L Walters RG Kutalik Z Martinet D Shen Y Valsesia A Beckmann ND Thorleifsson G Belfiore M Bouquillon S Campion D de Leeuw N de Vries BB Esko T Fernandez BA Fernández-Aranda F Fernández-Real JM Gratacòs M Guilmatre A Hoyer J Jarvelin MR Kooy RF Kurg A Le Caignec C Männik K Platt OS Sanlaville D Mirror extreme BMI phenotypes associated with gene dosage at the chromosome 16p11.2 locus Nature 2011 478 97 102 10.1038/nature10406 21881559 PMC3637175

[B9] Walters RG Jacquemont S Valsesia A de Smith AJ Martinet D Andersson J Falchi M Chen F Andrieux J Lobbens S Delobel B Stutzmann F El-Sayed Moustafa JS Chèvre JC Lecoeur C Vatin V Bouquillon S Buxton JL Boute O Holder-Espinasse M Cuisset JM Lemaitre MP Ambresin AE Brioschi A Gaillard M Giusti V Fellmann F Ferrarini A Hadjikhani N Campion D A new highly penetrant form of obesity due to deletions on chromosome 16p11.2 Nature 2010 463 671 675 10.1038/nature08727 20130649 PMC2880448

[B10] Jarick I Vogel CI Scherag S Schäfer H Hebebrand J Hinney A Scherag A Novel common copy number variation for early onset extreme obesity on chromosome 11q11 identified by a genome-wide analysis Hum Mol Genet 2011 20 840 852 10.1093/hmg/ddq518 21131291 PMC3024044

[B11] Jamshidi Y Snieder H Ge D Spector TD O’Dell SD The SH2B gene is associated with serum leptin and body fat in normal female twins Obesity (Silver Spring) 2007 15 5 9 10.1038/oby.2007.637 17228025 PMC1780257

[B12] Haupt A Thamer C Heni M Machicao F Machann J Schick F Stefan N Fritsche A Häring HU Staiger H Novel obesity risk loci do not determine distribution of body fat depots: a whole-body MRI/MRS study Obesity (Silver Spring) 2010 18 1212 1217 10.1038/oby.2009.413 19910938

[B13] Hotta K Kitamoto T Kitamoto A Mizusawa S Matsuo T Nakata Y Hyogo H Ochi H Kamohara S Miyatake N Kotani K Komatsu R Itoh N Mineo I Wada J Yoneda M Nakajima A Funahashi T Miyazaki S Tokunaga K Masuzaki H Ueno T Chayama K Hamaguchi K Yamada K Hanafusa T Oikawa S Yoshimatsu H Sakata T Tanaka K Matsuzawa Y Nakao K Sekine A Computed tomography analysis of the association between the SH2B1 rs7498665 single-nucleotide polymorphism and visceral fat area J Hum Genet 2011 56 716 719 10.1038/jhg.2011.86 21796141

[B14] Ren D Li M Duan C Rui L Identification of SH2-B as a key regulator of leptin sensitivity, energy balance, and body weight in mice Cell Metab 2005 2 95 104 10.1016/j.cmet.2005.07.004 16098827

[B15] Ren D Zhou Y Morris D Li M Li Z Rui L Neuronal SH2B1 is essential for controlling energy and glucose homeostasis J Clin Invest 2007 117 397 406 10.1172/JCI29417 17235396 PMC1765516

[B16] Morris DL Cho KW Rui L Critical role of the Src homology 2 (SH2) domain of neuronal SH2B1 in the regulation of body weight and glucose homeostasis in mice Endocrinology 2010 151 3643 3651 10.1210/en.2010-0254 20484460 PMC2940518

[B17] Yoganathan P Karunakaran S Ho MM Clee SM Nutritional regulation of genome-wide association obesity genes in a tissue-dependent manner Nutr Metab (Lond) 2012 1 65 22781276 10.1186/1743-7075-9-65 PMC3537611

[B18] Gutierrez-Aguilar R Kim DH Woods SC Seeley RJ Expression of new loci associated with obesity in diet-induced obese rats: from genetics to physiology Obesity (Silver Spring) 2012 2 306 312 21779089 10.1038/oby.2011.236

[B19] Bonnefond A Clément N Fawcett K Yengo L Vaillant E Guillaume JL Dechaume A Payne F Roussel R Czernichow S Hercberg S Hadjadj S Balkau B Marre M Lantieri O Langenberg C Bouatia-Naji N Charpentier G Vaxillaire M Rocheleau G Wareham NJ Sladek R McCarthy MI Dina C Barroso I Jockers R Froguel P The Meta-Analysis of Glucose and Insulin-Related Traits Consortium (MAGIC) Rare MTNR1B variants impairing melatonin receptor 1B function contribute to type 2 diabetes Nat Genet 2012 doi:10.1038/ng.1053 10.1038/ng.1053 PMC3773908 22286214

[B20] Hinney A Nguyen TT Scherag A Friedel S Brönner G Müller TD Grallert H Illig T Wichmann HE Rief W Schäfer H Hebebrand J Genome wide association (GWA) study for early onset extreme obesity supports the role of fat mass and obesity associated gene (FTO) variants PLoS One 2007 2 e1361 10.1371/journal.pone.0001361 18159244 PMC2137937

[B21] Scherag A Dina C Hinney A Vatin V Scherag S Vogel CI Müller TD Grallert H Wichmann HE Balkau B Heude B Jarvelin MR Hartikainen AL Levy-Marchal C Weill J Delplanque J Körner A Kiess W Kovacs P Rayner NW Prokopenko I McCarthy MI Schäfer H Jarick I Boeing H Fisher E Reinehr T Heinrich J Rzehak P Berdel D Two new Loci for body-weight regulation identified in a joint analysis of genome-wide association studies for early-onset extreme obesity in French and german study groups PLoS Genet 2010 6 e1000916 10.1371/journal.pgen.1000916 20421936 PMC2858696

[B22] Hebebrand J Heseker H Himmelmann GW Schäfer H Remschmidt H Altersperzentilen für den Body Mass Index aus Daten der Nationalen Verzehrstudie einschließlich einer Übersicht zu relevanten Einflussfaktoren Aktuel Ernährungsmed 1994 19 259 265

[B23] Hinney A Bettecken T Tarnow P Brumm H Reichwald K Lichtner P Scherag A Nguyen TT Schlumberger P Rief W Vollmert C Illig T Wichmann HE Schäfer H Platzer M Biebermann H Meitinger T Hebebrand J Prevalence, spectrum, and functional characterization of melanocortin-4 receptor gene mutations in a representative population-based sample and obese adults from Germany J Clin Endocrinol Metab 2006 91 1761 1769 10.1210/jc.2005-2056 16492696

[B24] Reinehr T Hinney A de Sousa G Austrup F Hebebrand J Andler W Definable somatic disorders in overweight children and adolescents J Pediatr 2007 150 618 622 10.1016/j.jpeds.2007.01.042 17517246

[B25] Bau AM Ernert A Schenk L Wiegand S Martus P Grüters A Krude H Is there a further acceleration in the age at onset of menarche? A cross-sectional study in 1840 school children focusing on age and bodyweight at the onset of menarche Eur J Endocrinol 2009 160 107 113 18974233 10.1530/EJE-08-0594

[B26] Wabitsch M Hauner H Hertrampf M Muche R Hay B Mayer H Kratzer W Debatin KM Heinze E Type II diabetes mellitus and impaired glucose regulation in Caucasian children and adolescents with obesity living in Germany Int J Obes Relat Metab Disord 2004 28 307 313 14724655 10.1038/sj.ijo.0802555

[B27] Wichmann HE Gieger C Illig T MONICA/KORA Study Group KORA-gen—resource for population genetics, controls and a broad spectrum of disease phenotypes Gesundheitswesen 2005 67 Suppl 1 S26 30 16032514 10.1055/s-2005-858226

[B28] Nagel G Wabitsch M Galm C Berg S Brandstetter S Fritz M Klenk J Peter R Prokopchuk D Steiner R Stroth S Wartha O Weiland SK Steinacker J Determinants of obesity in the Ulm Research on Metabolism, Exercise and Lifestyle in Children (URMEL-ICE) Eur J Pediatr 2009 10 1259 1267 19562371 10.1007/s00431-009-1016-y

[B29] Hinney A Hohmann S Geller F Vogel C Hess C Wermter AK Brokamp B Goldschmidt H Siegfried W Remschmidt H Schäfer H Gudermann T Hebebrand J Melanocortin-4 receptor gene: case–control study and transmission disequilibrium test confirm that functionally relevant mutations are compatible with a major gene effect for extreme obesity J Clin Endocrinol Metab 2003 88 4258 4267 10.1210/jc.2003-030233 12970296

[B30] Carbonell P Turpin MC Torres-Moreno D Molina-Martínez I García-Solano J Perez-Guillermo M Conesa-Zamora P Comparison of allelic discrimination by dHPLC, HRM, and TaqMan in the detection of BRAF mutation V600E J Mol Diagn 2011 13 467 473 10.1016/j.jmoldx.2011.03.009 21708284 PMC3157612

[B31] Hinney A Schmidt A Nottebom K Heibült O Becker I Ziegler A Gerber G Sina M Görg T Mayer H Siegfried W Fichter M Remschmidt H Hebebrand J Several mutations in the melanocortin-4 receptor gene including a nonsense and a frameshift mutation associated with dominantly inherited obesity in humans J Clin Endocrinol Metab 1999 84 1483 1486 10.1210/jc.84.4.1483 10199800

[B32] Purcell S Neale B Todd-Brown K Thomas L Ferreira MA Bender D Maller J Sklar P de Bakker PI Daly MJ Sham PC PLINK: a tool set for whole-genome association and population-based linkage analyses Am J Hum Genet 2007 81 559 575 10.1086/519795 17701901 PMC1950838

[B33] Ramensky V Bork P Sunyaev S Human non-synonymous SNPs: server and survey Nucleic Acids Res 2002 30 3894 3900 10.1093/nar/gkf493 12202775 PMC137415

[B34] Bromberg Y Rost B SNAP: predict effect of non-synonymous polymorphisms on function Nucleic Acids Res 2007 35 3823 3835 10.1093/nar/gkm238 17526529 PMC1920242

[B35] Ferrer-Costa C Orozco M de la Cruz X Sequence-based prediction of pathological mutations Proteins 2004 57 811 819 10.1002/prot.20252 15390262

[B36] Schwarz JM Rödelsperger C Schuelke M Seelow D MutationTaster evaluates disease-causing potential of sequence alterations Nat Methods 2010 7 575 576 10.1038/nmeth0810-575 20676075

[B37] Rosenblum CI Vongs A Tota MR Varnerin JP Frazier E Cully DF Morsy MA Van der Ploeg LH A rapid, quantitative functional assay for measuring leptin Mol Cell Endocrinol 1998 143 117 123 10.1016/S0303-7207(98)00129-4 9806356

[B38] Li Z Zhou Y Carter-Su C Myers MG Rui L Jr SH2B1 enhances leptin signaling by both Janus kinase 2 Tyr813 phosphorylation-dependent and –independent mechanisms Mol Endocrinol 2007 21 2270 2281 10.1210/me.2007-0111 17565041

[B39] Qian X Ginty DD SH2-B and APS are multimeric adapters that augment TrkA signaling Mol Cell Biol 2001 21 1613 1620 10.1128/MCB.21.5.1613-1620.2001 11238898 PMC86707

[B40] Morris DL Rui L Recent advances in understanding leptin signalling and leptin resistance Am J Physiol Endocrinol Metab 2009 297 E1247 1259 10.1152/ajpendo.00274.2009 19724019 PMC2793049

[B41] Morris DL Cho KW Zhou Y Rui L SH2B1 enhances insulin sensitivity by both stimulating the insulin receptor and inhibiting tyrosine dephosphorylation of insulin receptor substrate proteins Diabetes 2009 58 2039 2047 10.2337/db08-1388 19542202 PMC2731532

[B42] Duan C Li M Rui L SH2-B promotes insulin receptor substrate 1 (IRS1)- and IRS2-mediated activation of the phosphatidylinositol 3-kinase pathway in response to leptin J Biol Chem 2004 279 43684 43691 10.1074/jbc.M408495200 15316008 PMC3874232

[B43] Zhang M Deng Y Riedel H PSM/SH2B1 splice variants: critical role in src catalytic activation and the resulting STAT3s-mediated mitogenic response J Cell Biochem 2008 104 105 118 10.1002/jcb.21606 18247337

[B44] Geller F Reichwald K Dempfle A Illig T Vollmert C Herpertz S Siffert W Platzer M Hess C Gudermann T Biebermann H Wichmann HE Schäfer H Hinney A Hebebrand J Melanocortin-4 receptor gene variant I103 is negatively associated with obesity Am J Hum Genet 2004 74 572 581 10.1086/382490 14973783 PMC1193776

[B45] Stutzmann F Vatin V Cauchi S Morandi A Jouret B Landt O Tounian P Levy-Marchal C Buzzetti R Pinelli L Balkau B Horber F Bougnères P Froguel P Meyre D Non-synonymous polymorphisms in melanocortin-4 receptor protect against obesity: the two facets of a Janus obesity gene Hum Mol Genet 2007 16 1837 1844 10.1093/hmg/ddm132 17519222

[B46] Xiang Z Litherland SA Sorensen NB Proneth B Wood MS Shaw AM Millard WJ Haskell-Luevano C Pharmacological characterization of 40 human melanocortin-4 receptor polymorphisms with the endogenous proopiomelanocortin-derived agonists and the agouti-related protein (AGRP) antagonist Biochemistry 2006 45 7277 88 10.1021/bi0600300 16752916

[B47] Doche ME Bochukova EG Su HW Pearce LR Keogh JM Henning E Cline JM Dale A Cheetham T Barroso I Argetsinger LS O'Rahilly S Rui L Carter-Su C Farooqi IS Human SH2B1 mutations are associated with maladaptive behaviors and obesity J Clin Invest 2012 122 4732 4736 10.1172/JCI62696 23160192 PMC3533535

