# Supplementary material for: Mutation screen in the GWAS derived obesity gene SH2B1 including functional analyses of detected variants
Source: BMC Med Genomics. 2012 Dec 27;5:65. doi: 10.1186/1755-8794-5-65 (PMC3544595; doi:10.1186/1755-8794-5-65)
Supplement: Additional file 1 — Table 1.In silico functional prediction of detected non-synonymous mutations in SH2B1.Table2: Parameters of leptin receptor activity measured by STAT3 mediated luciferase response. Figure1: Identified variants in the three splice variants (α, β and γ) of human SH2B1. SH2B1 mRNA – coding parts as filled blocks – (Ensembl sequences α: ENST00000322610, β: ENST00000359285 and γ: ENST00000337120). The domain structure (Quian and Ginty 2001) with dimerization, Pleckstrin homology and SH2 domain is shown as underlying grey boxes. Positions of detected variants are marked with lines. Available rs-numbers, if applicable amino acid exchanges and minor allele frequencies in obese cases (MAF according to Table1) are given for each variant. Supplementary material: In silico analysis tool description. Supplementary Figure2: Regional association and linkage disequilibrium plot of 1000 genome project data centered to SNP rs7498665 (http://www.525broadinstitute.org/mpg/snap/). Displayed are recombination rate (blue), r² to rs7498665 (range of grey, increased intensity shows higher linkage) and genes in region. Dashed lines mark a region in high LD (r² > 0.8) with rs7498665. Gene abbreviations: EIF3CL/EIF3C (eukaryotic translation initiation factor 3), CLN3 (ceroid-lipofuscinosis, neuronal 3), APOB48R (apolipoprotein B48 receptor), IL27 (interleukin 27), NUPR1 (p8 protein isoform a), CCDC101 (coiled-coil domain containing 101), SULT1A1 (sulfotransferase family, cytosolic, 1A, member 1), SULT1A2 (sulfotransferase family, cytosolic, 1A,member 2), ATXN2L (ataxin 2 related protein isoform C), TUFM (Tu translation elongation factor, mitochondrial), SH2B1 (SH2B adaptor protein 1 isoform 1), ATP2A1 (ATPase, Ca++ transporting, fast twitch 1 isoform), RABEP2 (rabaptin, RAB GTPase binding effector protein 2), CD19 (CD19 antigen precursor), NFATC2IP (Nuclear factor of activated T-cells, cytoplasmic 2-interacting protein), SPNS1 (spinster homolog 1 isoform 1), LAT (linker for activation of T cel [file 1755-8794-5-65-S1.docx]

**Supplementary Table 1: *In silico* functional prediction of detected non-synonymous mutations in *SH2B1***

|  |  | **PolyPhen-2** | | **SNAP** | | | **PMUT** | | | **Mutation Taster** | |  |
| --- | --- | --- | --- | --- | --- | --- | --- | --- | --- | --- | --- | --- |
| **Amino acid changes** | **DNA position** | *Delta_Score* | *Prediction* | *RI* | *Expected Accuracy (%)* | *Prediction* | *Score* | *Reliability* | *Prediction* | *Prediction* | *Probability* | **Conservation** |
| Thr175Asp | g.2749C/A | 0.000 | Benign | 0 | 53 | neutral | 0.2582 | 4 | neutral | neutral | 0.6181 | 71% |
| Thr484Ala | g.8164A/G | 0.219 | Benign | 4 | 85 | neutral | 0.3210 | 3 | neutral | neutral | 0.9999 | 5% |
| βThr656Ile | g.9483C/T | 0.107 | Benign | 1 | 63 | not neutral | 0.9386 | 8 | pathological | disease causing | 0.9992 | 86% |
| γPro674Ser | g.9483C/T | 0.038 | Benign | 0 | 53 | neutral | 0.2451 | 5 | neutral | disease causing | 0.9992 | 100% |

**PolyPhen-2** computes the absolute difference between profile scores (Delta_Score) of both allelic variants in the polymorphic position with a prediction of the functional outcome of the exchange. **SNAP** gives a reliability index (RI) that ranges from 0 (low) to 9 (high). Only results with an expected accuracy >50% are displayed. **PMUT** predictor gives a pathogenicity index (Score) ranging from 0 to 1 (an index > 0.5 signals pathological mutations) and a confidence index (Reliability) ranging from 0 (low) to 9 (high) were calculated. **Mutationtaster** uses a Bayes classifier to calculate probabilities if the alteration in the sequence is a disease mutation or a harmless polymorphism. A probability close to 1 indicates a high security of prediction.

Conservation was analyzed as percentage of species (given in the materials and methods section) carrying the same amino acid on the position of the exchange. As not all species express all splice variants, 21 species were analyzed for Thr175Asp and Thr484Ala, 8 species for βThr656Ile and 6 species for γPro674Ser (for details, see Methods).

**SupplementaryTable 2: Parameters of leptin receptor activity measured by STAT3 mediated luciferase response**

**

HEK293 cells (n=8 separate experiments) were co-transfected with *LEPRb*, a STAT3 responsive element and *SH2B1* splice variants beta (left) and gamma (right) with and without the infrequent alleles at rs7498665 (Thr484Ala) and *β*Thr656Ile/*γ*Pro674Ser. Depicted are basal activity without leptin treatment, maximal activation of the leptin receptor (Emax), and the half-maximal activation of the leptin receptor (EC50), of the leptin receptor co-transfected with clones of *SH2B1* harbouring the different variants; all with standard deviation (SD).

**Supplementary Figure 1**


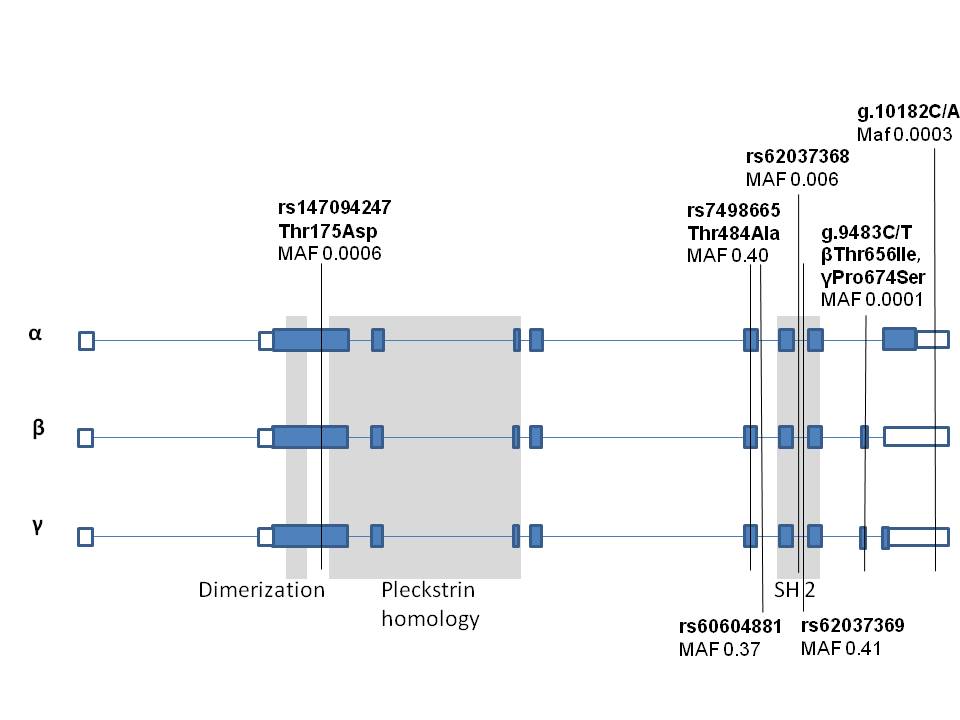


- Supplementary Figure 1 will be continued -
- Supplementary Figure 1 continued -

Identified variants in the three splice variants (α, β and γ) of human *SH2B1. SH2B1* mRNA – coding parts as filled blocks – (Ensembl sequences α: ENST00000322610, β: ENST00000359285 and γ: ENST00000337120). The domain structure (Quian and Ginty 2001) with dimerization, Pleckstrin homology and SH2 domain is shown as underlying grey boxes. Positions of detected variants are marked with lines. Available rs-numbers, if applicable amino acid exchanges and minor allele frequencies in obese cases (MAF **according to Table 1**) are given for each variant.**Supplementary material: *In silico* analysis tool description:**

To determine the potential alteration in gene expression, all mutations were analyzed for loss or gain of cryptic splice sites within the intronic region of *SH2B1* by ESEfinder (1), ESRsearch (2) and Rescue-ESE (3). Additionally, all mutations were screened for gain or loss of transcription factor binding sites via Tfsearch (4) and ConSite (http://asp.ii.uib.no:8090/cgi-bin/CONSITE/consite; 5), and gain or loss of o-glycosilation sites via OGPET (http://ogpet.utep.edu/OGPET/; 6).

Prediction of possible impact of amino acid exchange on structure and function of SH2B1 was done by PolyPhen-2 (Polymorphism Phenotyping-2; 7), SNAP (Screening for Non-Acceptable Polymorphisms; 8) PMUT (http://mmb2.pcb.ub.es:8080/PMut; 9) and MutationTaster (http://www.mutationtaster.org/; 10).

Conservation was analyzed by aligning sequences of 21 species in total (21 α, eight β and six γ sequences). Species were human (*Homo sapiens*; α NP_001139267.1, β NP_001139268.1, γ NP_001139269.1), chimp (*Pan troglodytes*; α ENSPTRP00000053639), gorilla (*Gorilla gorilla*; α ENSGGOP00000022688), mouse (*Mus musculus*; α NP_001074928.1, β NP_035493.2, γ AF421139, δ AF380422), rat (*Rattus norvegicus*; α NP_604451.2, β NP_001041645.1), horse (*Equus caballus*; α XP_003362765.1, β XP_001502284.1, γ XP_003362766.1), cattle (*Bos taurus*; α NP_001192458.1, β XP_872072.3), panda bear (*Ailuropoda melanoleuca*; α XP_002927372.1, β XP_002927373.1), Northern white-cheeked gibbon (*Nomascus leucogenys*; α XP_003261644.1, β XP_003261646.1, γ XP_003261648.1), Hoffmann’s Two-toed Sloth (*Choloepus hoffmanni*; β ENSCHOT00000007709), African bush elephant (*Loxodonta africana*; α ENSLAFP00000014023), Northern Treeshrew (*Tupaia belangeri*; α ENSTBEP00000013184), cat (*Felis catus*; α ENSFCAP00000002859), Large Flying Fox (*Pteropus vampyrus*; α ENSPVAP00000003750), Common Bottlenose Dolphin (*Tursiops truncatus*; α ENSTTRP00000007486), dog (*Canis lupus familiaris*; α, β, γ), Rhesus macaque (*Macaca mulatta*; α ENSMMUP00000030963, γ ENSMMUP00000030964), little brown bat (*Myotis Lucifugus*; α ENSMLUP00000009040), Nine-Banded Armadillo (*Dasypus novemcinctus*; α ENSDNOP00000000328), Rock Hyrax (*Procavia capensis*; α ENSPCAP00000014368) , common marmoset (*Callithrix jacchus*; α ENSCJAP00000011426, β ENSCJAP00000005535, γ ENSCJAP00000011454), guinea pig (*Cavia porcellus*; α ENSCPOP00000007490), pig (*Sus scrofa*; α ENSSSCP00000008333).

**References**

1. Cartegni L, Wang J, Zhu Z, Zhang MQ, Krainer AR: **ESEfinder: a web resource to identify exonic splicing enhancers.** *Nucleic Acid Res* 2003, **31**: 3568-3571.
2. Goren A, Ram O, Amit M, Keren H, Lev-Maor G, Vig I, Pupko T, Ast G: **Comparative analysis identifies exonic splicing regulatory sequences—The complex definition of enhancers and silencers.** *Mol Cell* 2006, **22**:769-781.
3. Fairbrother WG, Yeh RF, Sharp PA, Burge CB: **Predictive identification of exonic splicing enhancers in human genes.** Science 2002, **297**:1007-1013.
4. Heinemeyer T, Wingender E, Reuter I, Hermjakob H, Kel AE, Kel OV, Ignatieva EV, Ananko EA, Podkolodnaya OA, Kolpakov FA, Podkolodny NL, Kolchanov NA: **Databases on Transcriptional Regulation: TRANSFAC, TRRD, and COMPEL.** *Nucleic Acids Res* 1998, **26**: 364-370.
5. Sandelin A, Wasserman WW, Lenhard B**:** **ConSite: web-based prediction of regulatory elements using cross-species comparison.** *Nucleic Acids Res* 2004 **32**:W249-252.
6. Torres R, Almeida IC: **O-glycosylation Prediction Electronic Tool (OGPET): a new algorithm for prediction of O-glycosylation sites.** *FASEB J* 2006, **20**:1362.
7. Ramensky V, Bork P, Sunyaev S: **Human non-synonymous SNPs: server and survey.** *Nucleic Acids Res* 2002, **30**:3894-3900.
8. Bromberg Y, Rost B: **SNAP: predict effect of non-synonymous polymorphisms on function.** *Nucleic Acids Res* 2007, **35**:3823-3835.
9. Ferrer-Costa C, Orozco M, de la Cruz X: **Sequence-based prediction of pathological mutations**. *Proteins* 2004, **57**:811-819.
10. Schwarz JM, Rödelsperger C, Schuelke M, Seelow D: **MutationTaster evaluates disease-causing potential of sequence alterations.** *Nat Methods* 2010, **7**:575-576.

**Supplementary Figure 2**

- Supplementary Figure 2 will be continued -
- Supplementary Figure 2 continued -

Regional association and linkage disequilibrium plot of 1000 genome project data centered to SNP rs7498665 (http://www.broadinstitute.org/mpg/snap/). Displayed are recombination rate (blue), r² to rs7498665 (range of grey, increased intensity shows higher linkage) and genes in region. Dashed lines mark a region in high LD (r² > 0.8) with rs7498665. Gene abbreviations: *EIF3CL/EIF3C* (eukaryotic translation initiation factor 3), *CLN3* (ceroid-lipofuscinosis, neuronal 3), *APOB48R* (apolipoprotein B48 receptor), *IL27* (interleukin 27), *NUPR1* (p8 protein isoform a), *CCDC101* (coiled-coil domain containing 101), *SULT1A1* (sulfotransferase family, cytosolic, 1A, member 1), *SULT1A2* (sulfotransferase family, cytosolic, 1A,member 2), *ATXN2L* (ataxin 2 related protein isoform C), *TUFM* (Tu translation elongation factor, mitochondrial), *SH2B1* (SH2B adaptor protein 1 isoform 1), *ATP2A1* (ATPase, Ca++ transporting, fast twitch 1 isoform), *RABEP2* (rabaptin, RAB GTPase binding effector protein 2), *CD19* (CD19 antigen precursor), *NFATC2IP* (Nuclear factor of activated T-cells, cytoplasmic 2-interacting protein), *SPNS1* (spinster homolog 1 isoform 1), *LAT* (linker for activation of T cells isoform b)

**Supplementary Figure 3**

**
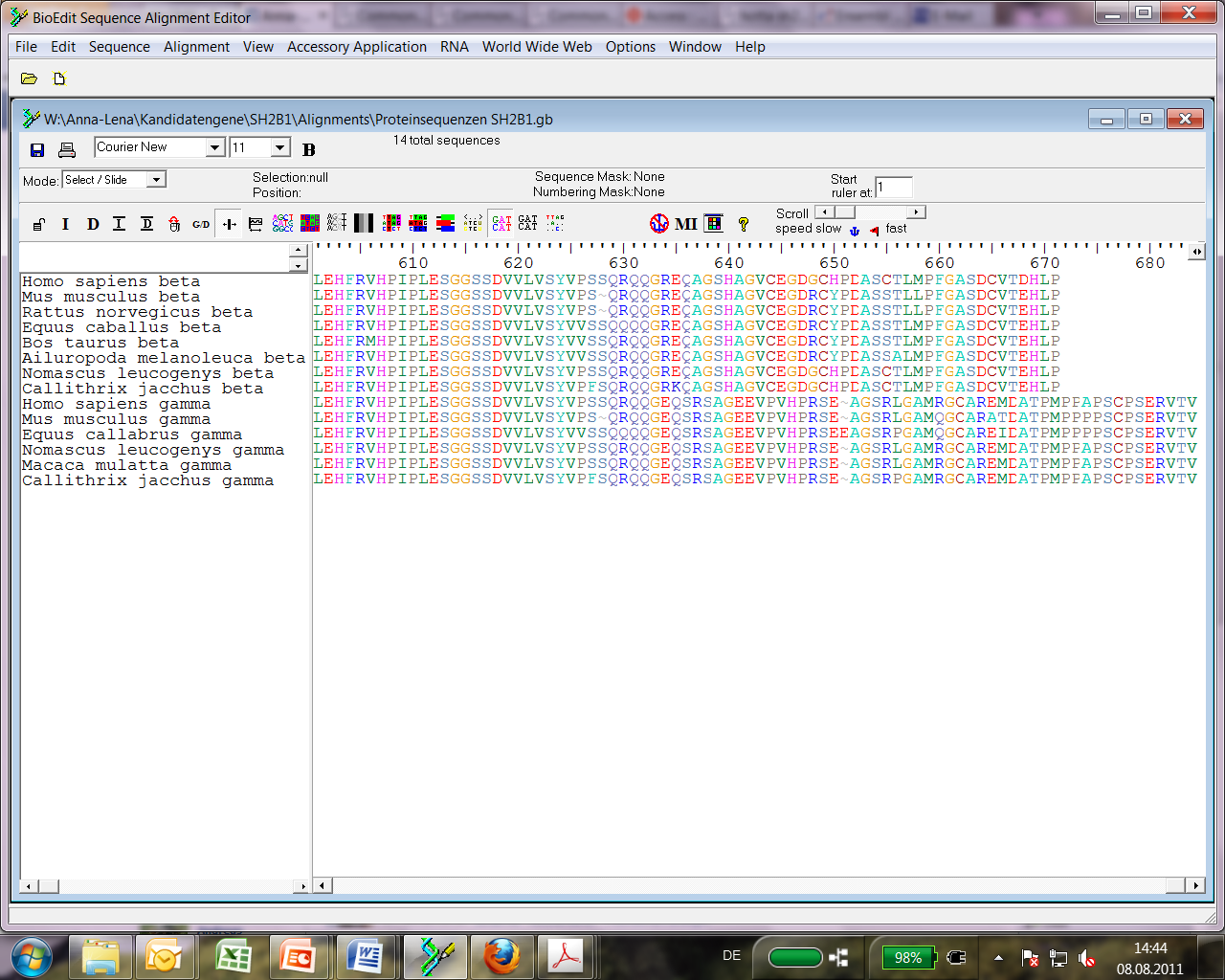
**

Conservation in C-terminal sequences of SH2B1 splice variants (β and γ). Boxes mark the position of exchange g.9483C/T (*β*Thr656Ile/*γ*Pro674Ser) in β and γ splice variants in several species.
